# Development of Extrudable Hydrogels Based on Carboxymethyl Cellulose–Gelatin Complex Coacervates

**DOI:** 10.3390/gels11010051

**Published:** 2025-01-08

**Authors:** Hamid Gharanjig, Hossein Najaf Zadeh, Campbell Stevens, Pram Abhayawardhana, Tim Huber, Ali Reza Nazmi

**Affiliations:** 1 School of Product Design, University of Canterbury, Christchurch 8041, New Zealandcampbell.stevens@canterbury.ac.nz (C.S.); pram.abhayawardhana@canterbury.ac.nz (P.A.); alireza.nazmi@canterbury.ac.nz (A.R.N.); 2 Biomolecular Interaction Centre, University of Canterbury, Private Bag 4800, Christchurch 8140, New Zealand; 3 Luxembourg Institute of Science and Technology, 5 Av. des Hauts-Fourneaux, 4362 Luxembourg, Luxembourg; tim.huber@list.lu

**Keywords:** hydrogel, coacervate gel, rheology, 3D extrusion printing, cryogel

## Abstract

This study investigates the 3D extrusion printing of a carboxymethyl cellulose (CMC)–gelatin complex coacervate system. Various CMC–gelatin coacervate hydrogels were prepared and analyzed to achieve this goal. The impact of the CMC–gelatin ratio, pH, and total biopolymer concentration on coacervation formation and rheological properties was evaluated to characterize the printability of the samples. Turbidity results indicated that the molecular interactions between gelatin and CMC biopolymers are significantly pH-dependent, occurring within the range of pH 3.7 to pH 5.6 for the tested compositions. Confocal Laser Scanning Microscopy (CLSM) confirmed the presence of coacervates as spherical particles within the optimal coacervation range. Scanning electron microscopy micrographs supported the CLSM findings, revealing greater porosity within this optimal pH range. Rheological characterization demonstrated that all CMC–gelatin hydrogels exhibited pseudoplastic behavior, with an inverse correlation between increased coacervation and decreased shear viscosity. Additionally, the coacervates displayed lower tackiness compared to gelatin hydrogels, with the maximum tackiness normal force for various CMC–gelatin ratios ranging from 1 to 15 N, notably lower than the 29 N observed for gelatin hydrogels. Mixtures with CMC–gelatin ratios of 1:15 and 1:20 exhibited the best shear recovery behavior, maintaining higher strength after shear load. The maximum strength of the CMC–gelatin coacervate system was found at a biopolymer concentration of 6%. However, lower biopolymer content allowed for consistent extrusion. Importantly, all tested samples were successfully extruded at 22 ± 2 °C, with the 1:15 biopolymer ratio yielding the most consistent printed quality. Our research highlights the promise of the CMC–gelatin coacervate system for 3D printing applications, particularly in areas that demand precise material deposition and adjustable properties.

## 1. Introduction

Complex coacervation system is a term used in colloid sciences that describes the process of associative phase separation caused by controlled modifications in environmental parameters, resulting in the formation of a coacervate phase [1] involving two or more polymers [2]. This process typically relies on electrostatic interactions between oppositely charged polymers [3]. Key factors influencing coacervation include the charge density of biopolymers, molecular weight distribution, biopolymer concentration, pH, ionic strength, temperature, and stirring rate [4,5]. While strong molecular interactions can lead to coacervate precipitation, moderate or weak interactions help maintain a stable coacervate phase.

Electrostatic interactions, particularly between anionic carbohydrates and proteins, are crucial in complex coacervation [6]. These interactions are sensitive to pH; proteins acquire a positive charge below their isoelectric point (pI), while polysaccharides typically remain negatively charged across a wide pH range. Gelatin type A has an isoelectric range typically between pH of 7 and 9. Below this range, the protein is positively charged, and its charge density increases with a further decrease in pH. Research has indicated that the zeta potential of gelatin type A approaches zero at pH 7 and gradually increases to about +15 mV at pH 2.5 [7,8]. In contrast, CMC with DS of 1.2 is a negatively charged polysaccharide, displaying a zeta potential of approximately −60 mV at pH values above 5, which drops significantly to around 0 at pH 2.0 [9]. The resulting ionic interactions between these two materials form particulate coacervates with light-scattering properties that increase turbidity as more particles are formed [10,11].

Polysaccharides, due to their versatility, biocompatibility, and biodegradability, are widely used in complex coacervate systems. Examples include gum Arabic, carrageenan, sodium carboxymethyl cellulose (CMC), hyaluronic acid (HA), hydroxypropyl cellulose (HPC), and modified curdlan. Some of the common proteins used in complex coacervates are whey protein [12], sesame protein [13], and gelatin [14]. The interaction between these biopolymers and their environmental conditions plays a significant role in determining the functional properties of complex coacervates.

There are various applications across different fields in which complex coacervates play crucial roles. Complex coacervation has been extensively applied in developing controlled-release carriers in pharmaceutical and drug delivery, the food industry [15], agriculture, and environmental applications [15,16]. Microencapsulation through coacervation provides a cost-effective, scalable method for incorporating bioactive ingredients with high reproducibility [15]. However, the unique rheological properties of complex coacervates and stabilization challenges have hindered their application in 3D printing [17,18].

The electrostatic molecular interactions within the biopolymers found in the coacervate phase can lead to distinct rheological properties, highly porous structures when freeze-dried, and improved mechanical characteristics [19]. This method has also been demonstrated to be effective for entrapping bioactive ingredients, thereby creating functional bioactive materials [2]. Investigations into rheology and efforts to develop extrudable hydrogels through complex coacervation have primarily focused on synthetic and chemically modified bio-based polymers, with very limited research conducted on natural biopolymers. As a result, further studies utilizing natural and commercially available biopolymers could broaden the biological applications of 3D-printable inks derived from complex coacervation.

Understanding the rheological requirements for 3D printing hydrogels is critical in addressing these challenges. 3D extrusion bioprinting, a subcategory of additive manufacturing (AM), enables the fabrication of shear-thinning materials into complex three-dimensional structures [20,21]. Suitable hydrogels require high viscosity, shear-thinning behavior for extrusion, and rapid structural recovery to maintain their shape [20,22]. Mixed gels, better referred to as yielding stress liquids, display enhanced viscoelastic properties compared to pure polysaccharide and protein solutions [23,24]. In complex coacervates, biopolymer characteristics, such as charge density, molecular weight, and functional groups, alongside environmental factors, such as pH and ionic strength, strongly influence extrudability [25].

The rheological behavior of soft materials often blurs the boundaries between gels and yielding stress liquids, making precise terminology essential for describing their properties. According to Malkin and Derkach (2024), a gel is defined as a solid, non-flowing substance characterized by a permanent three-dimensional network that immobilizes the liquid phase. In contrast, a yielding stress liquid exhibits solid-like behavior below a critical stress threshold (known as yield stress liquid) but flows like a liquid when the applied stress exceeds this threshold. This distinction is important because yielding stress liquids do not possess the permanent network found in gels; instead, they rely on weak, reversible supramolecular interactions or particle–particle associations that facilitate their transition from solid-like to liquid-like states [26]. In this study, we refer to a “CMC-gelatin coacervate system mixture” as a yielding stress liquid. This choice aims to avoid confusion and simplify the terminology for ease of use. Therefore, we use these terms interchangeably.

This study aims to introduce a new extrudable hydrogel based on complex coacervate, which is differentiated from hydrogels prepared using sole or multi biopolymer mixtures. Due to the lack of study in this area, coacervate gels were first prepared within the range of complex coacervation between carboxymethyl cellulose and gelatin type A. By optimizing critical coacervation parameters, such as pH, biopolymer ratios, and total biopolymer concentrations, this research addresses key challenges in stabilizing coacervates for 3D extrusion bioprinting. The findings hold significant potential for advancing active ingredient encapsulation in pharmaceuticals and nutraceuticals and contribute to developing sustainable and innovative solutions for various applications, such as food packaging.

## 2. Results and Discussion

### 2.1. Formation of Coacervate Gel: Effect of pH and CMC–Gelatin Ratio

**Figure 1:**
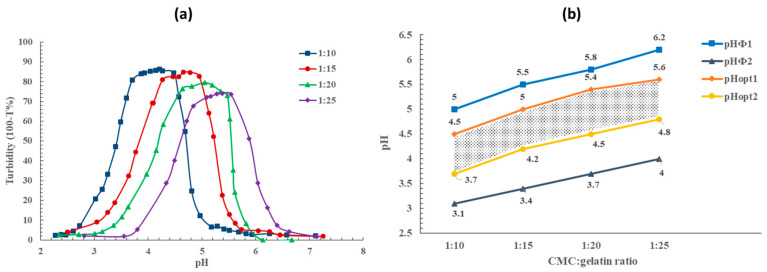
(**a**) Turbidity values as a function of pH for CMC–gelatin ratios of 1:10, 1:15, 1:20, and 1:25. (**b**) Coacervation pH window for CMC–gelatin ratios of 1:10, 1:15, 1:20, and 1:25. pH_Φ1_ and pH_Φ2_ represent subsets of dramatic increase or decrease in turbidity; pH_opt1_ and pH_opt2_ indicate the optimum pH range for coacervation.

Turbidity tests were conducted using various biopolymer ratios to determine the pH range at which coacervation occurs. Figure 1a illustrates the effect of the pH and the biopolymer ratio on turbidity. For all samples, turbidity peaked at a specific pH range and decreased when the pH was adjusted higher or lower. Coacervation was inhibited due to the reduction in the CMC charge by the protonation of anionic groups, resulting in a clear solution with a very low turbidity value. For a CMC–gelatin ratio of 1:10, the pH at which turbidity significantly increased was measured as 5.0 (referred to as pH_Φ1_ in Figure 1b). This value increased to 5.5, 5.8, and 6.2 for 1:15, 1:20, and 1:25 ratios. The second point of interest (pH_Φ2_), depicted in Figure 1a, shows a considerable decrease in turbidity as the coacervate particles dissolved. This was observed at a pH of 3.1 for the 1:10 ratio, pH 3.4 for the 1:15 ratio, pH 3.7 for the 1:20 ratio, and pH 4.0 for the 1:25 ratio. These findings demonstrate that increasing the gelatin content in the biopolymer coacervate system shifts the coacervation pH window to higher values. More gelatin in the coacervate system requires additional negatively charged polymeric chains to interact with the positive sites on the gelatin to form coacervates. Consequently, the ideal pH increases, allowing for a higher density of negative charge on the CMC molecules through less protonation of carboxylate groups [10]. Moreover, an increase in the gelatin content resulted in decreased turbidity, indicating that additional gelatin led to a lower coacervation yield, consistent with results reported by Duhoranimana et al. [27].

In addition to pH_Φ_, the optimum pH range for coacervation (pH_opt1_ and pH_opt2_) is illustrated in Figure 1b. This range is defined as the pH values where turbidity exceeds 95% of the maximum turbidity. The variation of turbidity within this optimum range is minimal. Within this range, biopolymers are electrostatically bonded, and the net charge is at its lowest level [15]. Previous studies have shown that the optimum coacervation pH is highly dependent on the grade of CMC (including DS and molecular weight), the type of gelatin, the biopolymer ratio, and ionic strength [27,28,29,30]. The turbidity results indicated that the molecular interactions between gelatin and CMC biopolymers are strongly influenced by the pH. The effect of this parameter on the coacervate mixture’s (yield stress liquid) strength and shear behavior was further investigated through rheological characterization.

**Figure 2:**
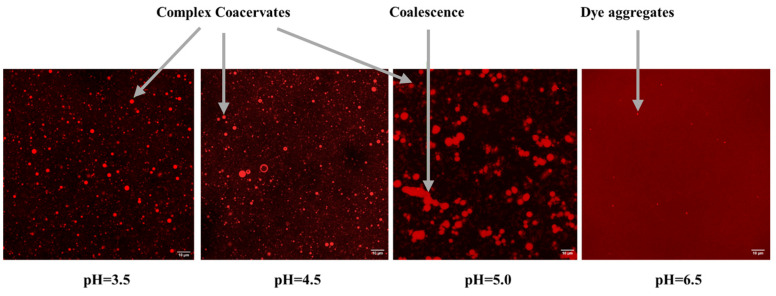
CLSM images of the CMC–gelatin mixture with a total biopolymer concentration of 0.1% (*w*/*w*) prepared at various pHs (the pH was gradually decreased through dropwise addition of acetic acid 50%, 10%, or 2% *w*/*v*) with gelatin stained using Rhodamine B (scale bar indicates 10 µm).

Confocal Laser Scanning Microscopy (CLSM) was employed to identify coacervate formation and visually investigate the structure of the CMC–gelatin coacervate system with a total biopolymer concentration of 0.1% (*w*/*w*) prepared at various pHs. The pH was gradually decreased through dropwise addition of acetic acid of 50%, 10%, or 2% *w*/*v*. As shown in Figure 2, coacervates appeared as spherical particles within the sample structure in samples prepared at pH 3.5, 4.5, and 5. Similar morphologies have been reported for complex coacervates [31]. No particles were detected in the sample prepared at pH 6.5 except for very bright points, which is likely due to dye aggregation, which were visible in all images. These findings confirm the absence of complex coacervation at pH 6.5, supporting the turbidity testing results. Additionally, at pH 5, which is within the optimum coacervation range, coacervates appeared larger in size, and particle coalescence was observed (as highlighted in the figure). At this pH, coacervates were distributed less uniformly, with a greater involvement of gelatin in their formation. As a result, fewer non-interacted gelatin molecules remained to create a strong and isotropic hydrogel.

**Figure 3:**
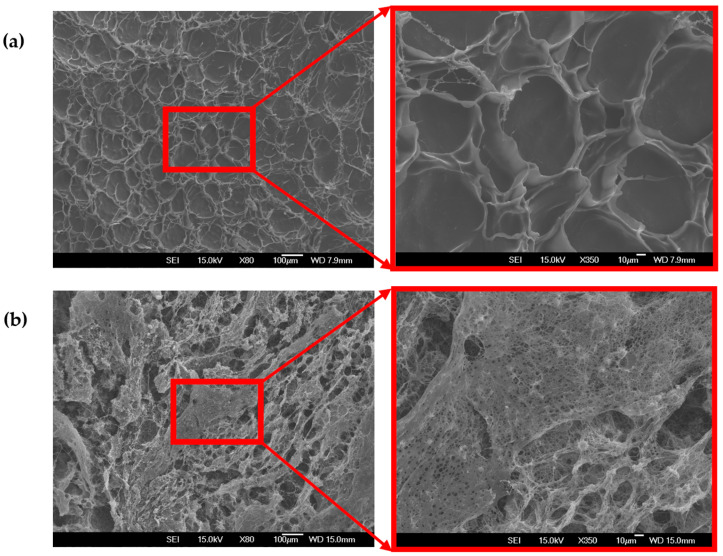
Micrograph of the CMC–gelatin cryogels prepared at a ratio of 1:20, a total biopolymer concentration of 4%, and different pHs: (**a**) pH 3.5 (outside of the coacervation range), (**b**) pH 5.0 (inside of the coacervation range).

As displayed in Figure 3, freeze-dried samples, called cryogels, exhibited a more sponge-like, porous structure. Figure 3a illustrates the less compact structure and larger pores of the cryogels prepared at pH 5.0 compared to samples prepared at pH 3.5, with high porosity and smaller micro-voids shown in Figure 3b. The formation of highly porous solid foams resulting from complex coacervation has been reported in several studies [19,32].

### 2.2. Rheological Properties: Effect of pH, CMC–Gelatin Ratio, and Total Biopolymer Concentration

The rheological behavior of CMC–gelatin coacervate system samples was analyzed to investigate the effects of the pH, biopolymer ratio, and total biopolymer concentration. These factors were considered to evaluate their potential as extrudable coacervate systems. Previous studies have shown that variables, such as the biopolymer ratio, the total biopolymer concentration, and the pH, significantly influence the intermolecular interactions between anionic polysaccharides and proteins during the formation of coacervate system samples [6,15,33]. These molecular interactions, primarily electrostatic interactions, can be modified in coacervate system by adjusting the abovementioned variables.

#### 2.2.1. Effect of pH on Rheological Properties

**Figure 4:**
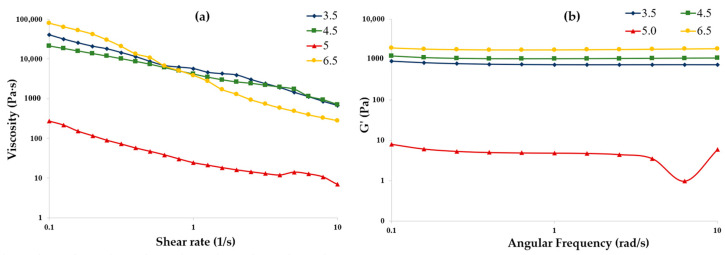
Steady-state viscosity (**a**) and storage modulus (**b**) for CMC–gelatin samples with a ratio of 1:20 and total biopolymer concentration of 4% at pH of 3.5 (♦), 4.5 (■), 5.0 (▲), and 6.5 (●).

The shear rheometric behaviors of CMC–gelatin samples at a 1:20 ratio and 4% biopolymer content at a pH of 3.5, 4.5, 5.0, and 6.5 at constant amplitude under increasing shear rate and angular frequency are shown in Figure 4a. The samples exhibited pseudoplastic behavior. Notably, the pH 6.5 sample demonstrated the largest viscosity at low shear rates. In contrast, samples prepared at a pH of 3.5, 4.5, and 5.0 showed lower viscosities at low shear rates. For all samples, shear rates exceeding 1 s^−1^ resulted in a decrease in apparent viscosity. This reduction is likely due to the applied forces overcoming the intra- and intermolecular forces within the polymer network, as well as the alignment of polymer chains in the direction of flow.

As shown in Figure 4a, the viscosity at shear rates below 1 s^−1^ is directly related to the formation of coacervates. Coacervates formed from CMC and gelatin at pH 5, which falls within the optimal range, exhibit the lowest viscosity. In contrast, samples at pH 3.5 and 4.5, which are near the edge of the coacervation range, show a noticeable increase in viscosity compared to the sample at pH 5. Meanwhile, samples at pH 6.5, which is outside of the optimal range for coacervation, demonstrate the highest viscosity at shear rates below 1 s^−1^.

Although CMC–gelatin samples at pH 6.5 present the highest viscosity, other coacervate system samples display pseudoplastic behavior and exhibit sufficient low shear rate viscosities, making them suitable for extrusion.

A similar trend was observed in the storage modulus (G′) of the samples, as illustrated in Figure 4b; the storage modulus increased as the sample pH moved outside of the coacervation range. The results indicate that a small change in pH from 4.5 to 5.0 resulted in a storage modulus that was nearly 150 times smaller when measured at 0.1 rad/s. This finding suggests that even slight variations in pH can lead to significant changes in the elastic properties and, consequently, the sample strength of the CMC–gelatin coacervate system.

Similarly to the viscosity results, the highest storage modulus (G′) was observed in the sample prepared at pH 6.5, which lies outside of the coacervation range. Conversely, the lowest storage modulus was seen in samples at pH 5.0 within the optimal coacervation range. This indicates that samples prepared at pH levels further outside of the coacervation range can achieve greater mechanical properties compared to those within it. This trend is also evident in samples prepared at pH 3.5 and pH 4.5.

It should be noted that coacervate systems differ from hydrogels made solely from gelatin or anionic polysaccharides. Coacervate systems form through the interaction of charged functional groups from different biopolymers, which become partially or fully neutralized by oppositely charged groups. As a result, pH plays a critical role in the stability of coacervate structures as it has a significant effect on biopolymer charge.

The complex coacervation between CMC and gelatin affects the structure of the mixed samples, resulting in reduced strength and lower shear rate viscosity. Within the coacervation range (pH 3.5, 4.5, and 5.0), gelatin carries a positive charge and interacts with CMC, leading to molecular deformation or conformational changes. The conformational changes of biopolymers due to complex coacervation have been confirmed in the literature using techniques like fluorometry, FTIR, and isothermal titration calorimetry [34,35,36]. Due to the interactions between gelatin and CMC, the protein does not expand enough to establish a strong gel network within the coacervate. This leads to the reduced viscosity and storage modulus observed in Figure 4. Furthermore, the stronger samples prepared at pH 6.5 should not be categorized as a coacervate system, as it was not formed within the range of complex coacervation.

#### 2.2.2. Effect of CMC–Gelatin Ratio on Rheological Properties

**Figure 5:**
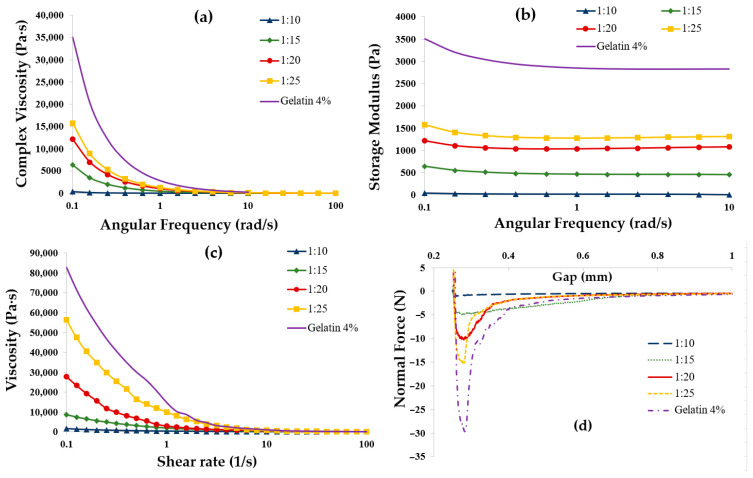
Rheological behavior of coacervate system samples at a concentration of 4% and CMC–gelatin ratio of 1:10 (▲), 1:15 (♦), 1:20 (●), and 1:25 (■) compared to gelatin at 4% concentration (Complex viscosity (**a**), Storage modulus (**b**), Controlled shear viscosity (**c**), and Tackiness (**d**)).

The impact of the CMC–gelatin biopolymer ratio on the rheological properties of coacervate system samples prepared at pH 4.5, with a total biopolymer concentration of 4%, was investigated. These coacervate system samples were then compared to samples made solely of 4% gelatin. The resulting rheograms are shown in Figure 5.

The complex viscosity and controlled shear viscosity (illustrated in Figure 5a and Figure 5c, respectively) indicate a decrease in viscosity with an increase in total CMC content. All coacervates exhibited shear-thinning behavior, showing a viscosity of 45 ± 15 Pa·s at a shear rate of 100 s^−1^. The viscosity measurements at a shear rate of 0.1 s^−1^ for coacervate samples with CMC–gelatin ratios of 1:25, 1:20, 1:15, and 1:10 were recorded as 5.6 × 10^4^, 2.8 × 10^4^, 8.6 × 10^3^, and 1.5 × 10^3^ Pa·s, respectively. Overall, all coacervate samples exhibited viscosity levels that were significantly lower than those of the gelatin alone.

Increasing the CMC concentration in CMC–gelatin coacervate system samples resulted in a lower storage modulus, as shown in Figure 5b. Specifically, the storage modulus at 0.1 rad/s decreased from 1574 Pa for a CMC–gelatin ratio of 1:25 to 36.5 Pa for a ratio of 1:10. For comparison, the storage modulus of pure gelatin was measured at 3.5 × 10^3^ Pa, which falls within the range reported for high molecular weight type-A gelatin derived from porcine skin [37]. Increasing the concentration of CMC leads to greater interaction between gelatin molecular chains and negatively charged polysaccharides. This interaction can hinder the formation of complexes and prevent the development of stronger networks. In other words, as the CMC content increases, the formation of bridged triple helical structures in gelatin is inhibited. Similar findings have been reported for complexes formed by gelatin interacting with chitosan and xanthan gum [23].

An increase in the concentration of CMC led to a reduction in the tackiness of the samples. The maximum normal forces detected for CMC–gelatin ratios of 1:10, 1:15, 1:20, and 1:25 were about 1 N, 5 N, 10 N, and 15 N, respectively. All of these values were significantly lower than that of 4% gelatin gels, which measured 29.66 N. Additionally, the values for storage modulus, complex viscosity, and viscosity over shear rate for coacervate samples were substantially lower than those of 4% gelatin. This suggests that even a small amount of CMC (3.8% of the total biopolymer weight) significantly altered the rheological properties of gelatin, making it easier to flow and more suitable for extrusion. However, the coacervate samples exhibited very weak yield stress liquid structures or low viscosities at low shear rates, which made it challenging to form extruded structures, particularly at lower ratios. A similar trend was observed for the impact of CMC–gelatin ratios at total biopolymer concentrations of 5% and 6% *w*/*w*.

**Figure 6:**
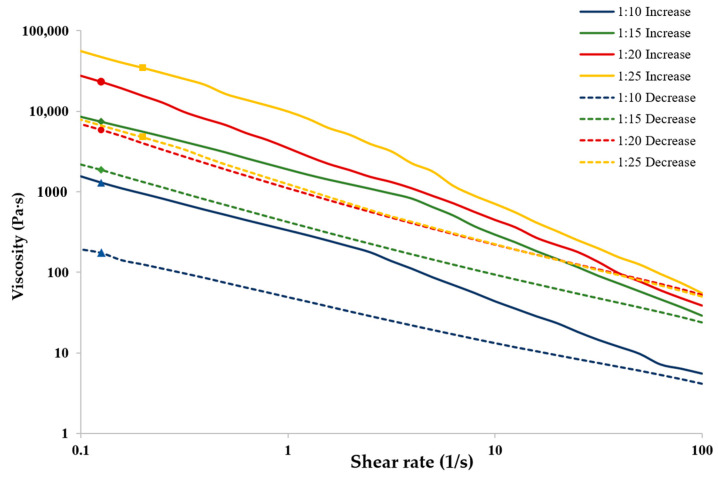
Steady-state-controlled shear rheometry for coacervate system samples prepared with CMC–gelatin ratios of 1:10 (▲), 1:15 (♦), 1:20 (●), and 1:25 (■) at a total biopolymer concentration of 4% and pH 4.5, showcasing behavior under increasing (solid line) and decreasing (dashed line) shear rates.

For a hydrogel, a gel, or a yield stress liquid to be suitable for extrusion, it must not only have sufficient low shear viscosity and exhibit pseudoplastic behavior but also possess thixotropic properties. This means it should quickly regenerate its structure after being extruded. Specifically, the hydrogel should recover a significant portion of its initial low shear viscosity after experiencing high shear loads. In this context, gelatin is not considered an ideal extrudable material because it shows minimal thixotropic properties. Figure 6 presents the controlled shear viscosity values obtained from a series of increasing and then decreasing shear rate ramps for coacervate samples made at a total biopolymer concentration of 4% and a pH of 4.5 (within the coacervation range of all samples) at CMC–gelatin ratios of 1:10, 1:15, 1:20, and 1:25. The samples with CMC–gelatin ratios of 1:15 and 1:20 demonstrated the smallest loss in shear viscosity, indicating the best structure recovery of all of the tested ratios. The percent change in viscosity for shear ramps from 1 s^−1^ to 100 s^−1^ and vice versa for ratios of 1:10, 1:15, 1:20, and 1:25 was −86.6%, −74.6%, −75.1%, and −86.0%, respectively. This indicates the effect of changing shear rates on coacervate system samples of various biopolymer ratios. Although none of the coacervate gels tested were able to return to their initial low shear viscosity at a shear rate of 0.1 s^−1^, all samples exhibited shear recovery characteristics suitable for extrusion.

#### 2.2.3. Effect of Biopolymer Concentration on Rheological Properties

**Figure 7:**
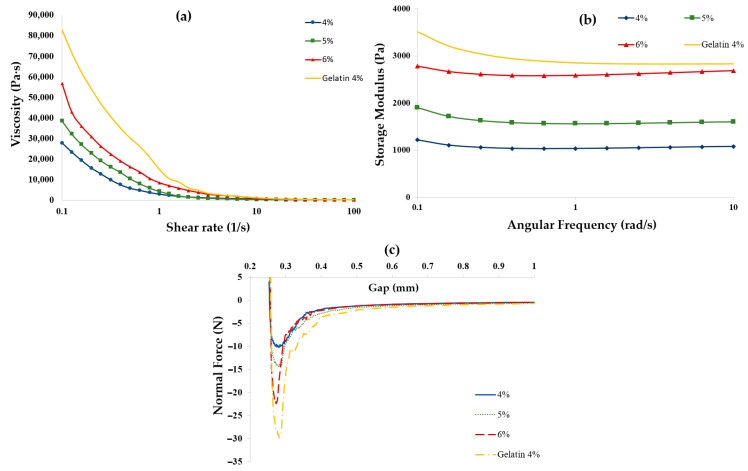
Rheological characteristics of coacervate system samples prepared at pH 4.5, CMC–gelatin ratio of 1:20, and total biopolymer concentration of 4% (●), 5% (■), and 6% (▲) and 4% sole gelatin hydrogel (Controlled shear viscosity (**a**), Storage Modulus (**b**), and Tackiness (**c**)).

Rheograms for coacervate system samples prepared at pH 4.5, with a CMC–gelatin ratio of 1:20 and total biopolymer concentrations of 4%, 5%, and 6% *w*/*w*, are presented in Figure 7, alongside results of 4% gelatin hydrogel. The viscosity and storage moduli of the samples increased with higher total biopolymer concentrations. At a total biopolymer concentration of 4%, the viscosity at a shear rate of 0.1 s^−1^ was measured at 2.8 × 10^4^ Pa·s. This value increased by factors of 1.38 and 2.04 when the total biopolymer concentration was raised to 5% and 6%, respectively. Additionally, the strength improved as the biopolymer concentration increased, with storage moduli rising from 1216.2 Pa at 4% concentration to 2785.9 Pa at 6% concentration.

The increase in sample strength and low shear viscosity with rising biopolymer concentration can be attributed to the presence of non-interacted gelatin, which facilitates the formation of a stronger three-dimensional network. This stronger network results in a more cohesive mixture with increased tackiness, as illustrated in Figure 7c. In tack experiments, the normal force required for the 6% total biopolymer concentration was significantly lower than that for the 4% gelatin alone, which is a positive indication of potential extrudability.

These findings suggest that incorporating CMC with gelatin enhances extrudability—characterized by reduced tack and more suitable low shear rate viscosity—at higher biopolymer concentrations compared to using gelatin alone, albeit at the expense of lower storage moduli. Although the 6% coacervate samples had reduced tack compared to the 4% gelatin samples, they still exhibited inconsistent deposition through the extrusion nozzle in comparison to 4% coacervate samples. This was due to high tack resulting in inconsistent flow, as the sample would stick to the extrusion nozzle. Further adjustments to the sample pH, the biopolymer concentration, and the CMC–gelatin ratio could be explored to develop a yield stress liquid with both high printability (appropriate tack and low shear rate viscosity) and greater strength (improved storage modulus through increased biopolymer concentration). However, exploring these adjustments was beyond the scope of this paper.

### 2.3. Assessing Extrudability of Coacervate System Mixtures

Coacervate system samples with CMC–gelatin ratios of 1:10, 1:15, 1:20, and 1:25 were prepared to assess extrudability, also referred to as printability. All samples contained 4% total biopolymer content, which was determined to be ideal for extrusion due to its optimal tack behavior and low shear rate viscosity. The pH of each sample was adjusted to fall within their optimal coacervation range, as described in Section 2.1, to ensure a high degree of biopolymer interactions.

The thermal properties of samples, including their average melting and re-solidification temperatures, are presented in Table 1. These values were determined using differential scanning calorimetry. These results indicated that the samples solidified at sub-ambient temperatures of approximately 12 °C and melted at 32 °C. The melting and solidification temperatures appear to be largely influenced by the gelatin content, which aligns with findings in the literature on gelatin’s thermal behavior [38,39]. While the inclusion of CMC and the formation of coacervation altered the phase change temperatures from previously reported values, the changes were minimal. Consequently, all samples could be extruded at approximately 22 °C, which is considered ambient temperature.

It was found that a significant factor affecting extrudability was gelatin swelling, a time-dependent phenomenon that occurs across all pH levels and temperatures ranging from 25 °C to 50 °C [40]. Greater swelling occurs at pH levels further from the sample’s isoelectric point, and swelling occurs most rapidly at temperatures between the mixture’s solidification and melting points [41,42]. Non-interacted gelatin within the coacervate solution would begin to swell as the sample cooled towards room temperature. This swelling leads to a more constrained polymer network due to increased gelatin crosslinking. This could result in reduced extrudability by increasing low shear rate viscosity and reducing shear recovery behavior. As a result, the samples have a specific “window of extrudability”, indicating the time frame within which they should be extruded before gelatin swelling begins. All samples were printed within this designated window, which was determined using a “tube inversion test”.

The tube inversion test, a qualitative method to assess the flow behavior of the coacervate solutions, was employed to identify the optimal “window of extrudability”. This simple test provided critical insights into the rheological properties of the samples, specifically indicating when the samples transitioned from a flowable state to a more constrained polymer network due to gelatin swelling. By observing the samples’ ability to flow when inverted, we established the approximate time frame (around two hours after reaching ambient temperature) during which the samples could be extruded effectively [43,44,45].

To validate these findings, additional printing tests were conducted to ensure that the identified extrudability window correlated with successful extrusion and consistent printing quality. These tests confirmed that samples printed within this window exhibited minimal issues related to swelling-induced viscosity changes and shear recovery behavior. This integration of qualitative (tube inversion) and quantitative (printing tests) approaches allowed us to establish practical guidelines for the extrusion process and highlight the time sensitivity associated with gelatin swelling in coacervate solutions.

**Figure 8:**
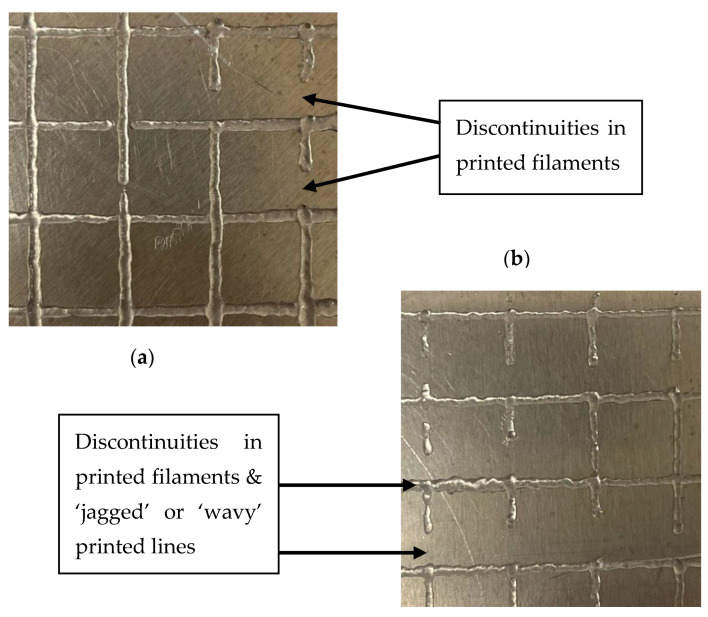
Print failures: examples of discontinuities in printed samples of (**a**) 1:20 ratio and (**b**) 1:25 ratio.

**Figure 9:**
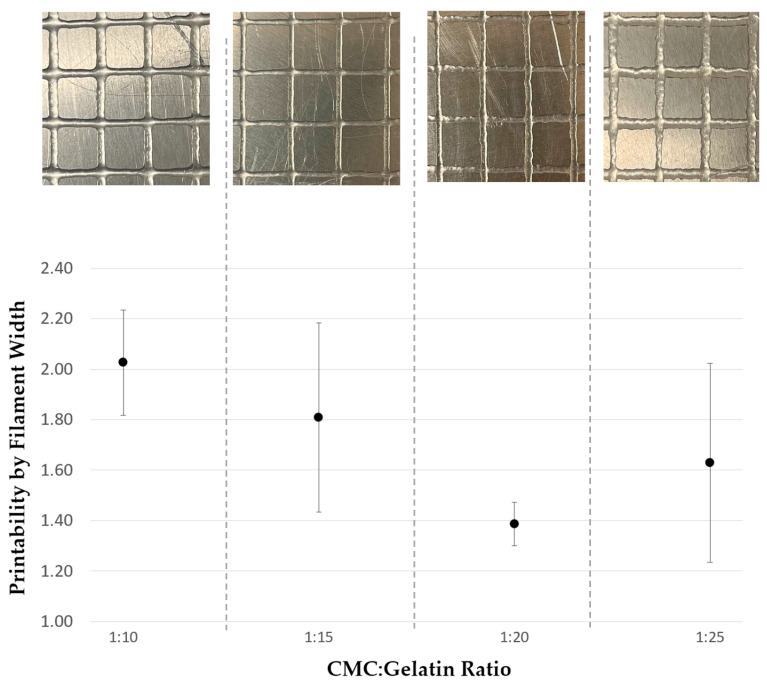
Printability of CMC–gelatin ratios 1:10. 1:15, 1:20, and 1:25, alongside images of their respective printed structures.

The parameters used for extruding the grid structure are shown in Table 2. For each sample, the pressure was initially set to 1.0 PSI. This pressure was incrementally increased until optimal flow behavior was observed for each sample. The nozzle speed was also adjusted between samples. After determining the optimal parameters, three repetitions of a grid pattern were extruded and analyzed. Examples of deposition failures are presented in Figure 8, while the measured printability of each sample, along with images of successful prints, is detailed in Figure 9. Printability is a common measure of how successfully materials are able to be extruded found by comparing the physical dimensions of an extruded shape to its intended dimensions. All prints shown in Figure 8 and Figure 9 were obtained using parameters detailed in Table 2.

All samples exhibited over-deposition of material, with the 1:10 ratio showing the largest line width. The CMC–gelatin ratio of 1:20 demonstrated the highest fidelity during 3D printing, achieving a printability value of 1.39. This was followed by 1:25, with a value of 1.63, 1:15 with 1.81, and 1:10 with 2.03. The 1:20 ratio also exhibited the lowest deviation in printability; its line thickness showed the least variation among all samples. Notably, the 1:15 ratio exhibited no discontinuities in its printed filaments, despite having the second-largest width deviation. In contrast, all other CMC–gelatin ratios showed discontinuities and other negative artifacts in their print filaments, as detailed in Figure 8. The 1:10 ratio displayed one minor discontinuity, 1:20 had five minor and one major discontinuities, and the 1:25 ratio had one minor and four major discontinuities. Minor discontinuities refer to one break per filament, while major discontinuities indicate two or more breaks per filament.

Variations in line width and discontinuities likely stem from non-interacted gelatin polymer deposits mixed within the coacervate phase. These deposits create variations in the shear behavior of the sample as it moves through the nozzle, resulting in inconsistent extrusion performance. Although this variation was not reflected in the printability value itself, it was visually evident, particularly in the 1:20 and 1:25 ratio samples, which displayed “jagged” or “wavy” lines, as shown in Figure 8. Despite their width variation and printing artifacts, all tested samples were successfully extruded, maintaining sufficient consistency to create a grid structure. The 1:15 ratio samples exhibited the most consistent print behavior within individual prints, with an acceptable line width, minimal “wavy” behavior, and zero discontinuities in all prints. The findings open avenues for exploring CMC–gelatin coacervate system mixtures/hydrogels in 3D printing applications, particularly in fields requiring precise deposition and tunable material properties.

## 3. Conclusions

This paper introduces an extrusion bioprintable coacervate system mixture/hydrogel based on CMC and gelatin complex coacervates, which have potential applications in active-loaded materials for biomedical use, drug delivery, and active packaging. Various CMC–gelatin coacervate system samples were prepared and analyzed for their rheological properties, demonstrating that these coacervates can be formulated into uniform mixtures/hydrogels that maintain suitable material properties in order to be extruded. Additionally, we investigated the effects of temperature on their flow behavior. An optimal ratio of 1:15 CMC–gelatin coacervate system was identified, which exhibited the highest fidelity in printed structures, and no discontinuities were observed during the printing process. This work enhances our understanding of gelatin and CMC interactions during coacervation and highlights the importance of the temperature, pH, biopolymer ratio, and total biopolymer content in achieving desirable viscosity and flow behavior for extrusion of biopolymers. Overall, our findings establish a foundation for future research in biopolymer applications and pave the way for innovative materials in 3D printing, with implications for tissue engineering, drug delivery, regenerative medicine, and active packaging.

## 4. Materials and Methods

### 4.1. Materials

Carboxymethyl cellulose (CMC) with a degree of substitution of 1.2 and an average molecular weight of 250,000 g∙mol^−1^ was purchased from Sigma Aldrich (St. Louis, MO, USA). Type-A gelatin from porcine skin with a gel strength of 300 bloom was purchased from Sigma Aldrich (St. Louis, MO, USA). Rhodamine B was purchased from Sigma Aldrich (St. Louis, MO, USA). Analytical-grade acetic acid and NaOH were purchased from Sigma Aldrich (St. Louis, MO, USA).

### 4.2. Preparation of CMC–Gelatin Mixtures/Hydrogels

Biopolymer stock solutions were prepared to obtain 2% and 10% *w*/*w* aqueous solutions of CMC and gelatin, respectively. Both solutions were prepared by slowly adding biopolymers to 50 °C deionized water under magnetic stirring at 800 RPM. Then, the solutions were vigorously mixed at 8000 rpm using a T25 Ultra Turrax (IKA, Staufen im Breisgau, Germany) for 30 s. Gelatin solution was used on the same day. CMC was stored in the fridge at 4 °C for 24 h for complete hydration and consumed within a month.

CMC–gelatin coacervate system mixtures were formed at various biopolymer concentrations, CMC–gelatin ratios, and pHs. Only the solid content within the solutions was considered when measuring the CMC–gelatin ratio and the total biopolymer concentrations. Freshly prepared gelatin solution was mixed with deionized water at 50 °C until thoroughly mixed. CMC solution was then added to this solution. After 15 min of mixing at 800 RPM, the pH of the mixture was adjusted using acetic acid (50%, 10%, and 2% *w*/*v*) or NaOH (0.5 M). Then, mixtures were vigorously mixed at 8000 rpm for 30 s. Resultant mixtures/gels were allowed to cool to ambient temperature (22 ± 2 °C).

### 4.3. Turbidity

Turbidity measurements were carried out on CMC–gelatin coacervate system mixtures to determine the complex coacervation pH range. An adequate quantity of CMC solution was mixed with deionized water and gelatin stock solution at 25 °C, and the pH was adjusted to 7 under continuous mixing for 15 min. CMC–gelatin coacervate system mixtures were prepared with a total biopolymer concentration of 0.1% (*w*/*w*). In the next step, the pH was gradually decreased through dropwise addition of acetic acid (50%, 10%, or 2% *w*/*v*). Higher concentrations of acetic acid were used for lower pHs to minimize the total volume change. Then, 4 mL of the mixture was poured into a cuvette, and the absorbance of the sample was read at 600 nm using a UV-1900 UV-VIS spectrophotometer (Shimadzu, Kyoto, Japan). At the same time, the pH of the sample was recorded using a pH 700 (Eutech Instruments, Singapore). The turbidity is defined as 100-T%, where T is the transmittance at 600 nm [27]. All measurements were made in triplicate.

### 4.4. Confocal Laser Scanning Microscope

Microstructural features of CMC–gelatin mixed samples were investigated using a TCS SP5 Confocal Laser Scanning Microscope (CLSM) (Leica, Germany). A 20× and 63× glycerol objective lens was used to observe the samples. Gelatin was stained with an aqueous solution of Rhodamine B under magnetic stirring for 30 min before mixing with CMC solutions. The dye binds non-covalently to the protein, and preliminary experiments showed that the addition of dye did not change the rheological behavior of the gels. CMC–gelatin mixed hydrogels were prepared with a 4% total biopolymer concentration, a CMC–gelatin ratio of 1:20, and pHs of 3.5, 4.5, 5, and 6.5. After mixing, the mixture was poured into FD35-100 Fluorodishes (World Precision Instrument, Inc., Sarasota, FL, USA), and samples were kept refrigerated at 4 °C for 24 h. Detection of stained gelatin was performed via excitation of rhodamine B at 550 nm, with emissions being recorded between 580 and 700 nm. Data were analyzed using the 5D Viewer plug-in of V1.51u ImageJ (FIJI, Madison, WI, USA).

### 4.5. Rheological Charactrization

Rheological measurements were carried out on an MCR 302 modular compact rheometer (Anton Paar, Graz, Austria). All measurements were conducted at 25 °C. Approximately 5 mL of the mixtures was poured onto the rheometer testing bed. The 50 mm Parallel Plate (CP50-1) was then lowered down to create a 1.025 mm gap. Excess material was carefully removed from the edges before lowering the plate further to create a 1.0 mm gap.

For steady-state-controlled shear viscometry, viscosity was measured by varying the shear rate from 0.1 to 100 s^−1^. Samples were then rested for 60 s, after which the same shear was applied to evaluate structure recovery. The power-law model was used to fit the experimental data and to determine the rheological parameters of coacervate gels.

Amplitude strain sweeps were applied with a range of 0.01 to 100% at a constant angular frequency (1 Hz) to determine the linear viscoelastic region (LVR).

Frequency sweep tests were performed with the constant strain (0.5%) within the LVR using a varying angular frequency from 0.1 to 100 rad/s.

Tack tests were performed by reducing the gap between the plates to 0.25 mm and raising the spindle at a rate of 100 µm∙s^−1^. The normal force to the spindle was measured for 500 points within 10 s of the test.

### 4.6. Scanning Electron Microscopy

Samples for SEM images were freeze-dried using a Labconco FreeZone freeze-dryer (Kanas City, MO, USA) at −50 °C and near vacuum for 48 h. Scanning electron microscopy (SEM) was used to observe the microstructure of the cryogels. The samples were attached to aluminum stubs using double-sided sticky tape and sputter-coated with a Quorum 150T-ES (East Sussex, UK) sputter coater under a vacuum with palladium. Coated cryogels were then observed using a SEM JEOL JSM-7000F (Tokyo, Japan) at an accelerating voltage of 15 kV.

### 4.7. Differential Scanning Calorimetry

Thermal analysis was performed on samples using a DSC8000 (Perkin Elmer, Waltham, MA, USA). Approximately 30 mg–40 mg of coacervate system mixture was encapsulated in small aluminum pans. These were heated from 5 °C to 50 °C at a rate of 10 °C/min. They were held at 50 °C for one minute. Samples were then cooled back to 5 °C at 10 °C/min before being held at that temperature for one more minute. The entire temperature loop was repeated twice to ensure that the thermal behavior was consistent. All tests were performed in triplicate, with unique samples used for each test.

### 4.8. 3D Extrusion Process

Coacervate system mixtures were prepared and transferred to printing barrels while hot and allowed to cool to room temperature (22 ± 2 °C) for 2 h. Barrels were fitted with 0.84 mm diameter (18 G) and 12.7 mm length metal tip nozzles. Printing was performed with a BioBot Basic bio-plotter (Advanced Solutions) (Louisville, KY, USA). Various 3D extrusion printing parameters (pressure, speed, line width, and height) were used to print a single-layer grid pattern. Print fidelity was assessed by comparing the line width of the printed filaments to the intended structure using similar methods to those recently described in the 3D bioprinting literature [46,47,48,49]. A measure of “Printability” was found by dividing the actual printed line width by the expected line width of 0.84 mm. Values closer to 1.0 were considered more successful. The assessment was performed using ImageJ software. Nine measures of line width were taken for each filament and averaged. An overall average of printability was then created from the average line widths of three printed grids for each composition. All printing trials were carried out using Tissue Structure Information Modelling (TSIM^®^) software version 1.1.227 by Advanced Solutions Life Sciences, (Louisville, KY, USA). All printing parameters (line width, line height, nozzle speed, nozzle acceleration, printing pressure, and printing temperature) are shown in Table 2.

## Figures and Tables

**Table 1:** Melting and solidification temperatures of various CMC–gelatin ratios.

CMC–Gelatin Ratio	Melting ±Deviation [°C]	Solidification ±Deviation [°C]
1:10	31.97 ± 0.54	12.12 ± 0.23
1:15	32.48 ± 0.01	12.48 ± 0.17
1:20	32.38 ± 0.39	12.17 ± 0.15
1:25	32.10 ± 0.55	12.81 ± 0.42

**Table 2:** Optimal printing parameters found for each CMC–gelatin ratio.

Printing Parameter	1:10	1:15	1:20	1:25
Line Width [mm]	0.84	0.84	0.84	0.84
Line Height [mm]	0.84	0.84	0.84	0.84
Nozzle Speed [mm/sec]	7.0	8.0	8.0	9.0
Nozzle Acceleration [mm/sec^2^]	6.0	6.0	6.0	6.0
Printing Pressure [PSI]	2.0	1.5	5.9	4.0
Temperature (°C)	22 ± 2	22 ± 2	22 ± 2	22 ± 2

## Data Availability

The data presented in this study are available upon request from the corresponding author.
